# Tin doped indium oxide anodes with artificially controlled nano-scale roughness using segregated Ag nanoparticles for organic solar cells

**DOI:** 10.1038/srep33533

**Published:** 2016-09-19

**Authors:** Hyo-Joong Kim, Eun-Hye Ko, Yong-Jin Noh, Seok-In Na, Han-Ki Kim

**Affiliations:** 1 Kyung Hee University, Department of Advanced Materials Engineering for Information and Electronics, 1 Seocheon, Yongin, Gyeonggi-do 446-701, Republic of Korea; 2 Chonbuk National University, Graduate School of Flexible and Printable Electronics, 664-14, Deokjin-dong, Jeonju-si, Jellabuk-do, 561-756, Republic of Korea

## Abstract

Nano-scale surface roughness in transparent ITO films was artificially formed by sputtering a mixed Ag and ITO layer and wet etching of segregated Ag nanoparticles from the surface of the ITO film. Effective removal of self-segregated Ag particles from the grain boundaries and surface of the crystalline ITO film led to a change in only the nano-scale surface morphology of ITO film without changes in the sheet resistance and optical transmittance. A nano-scale rough surface of the ITO film led to an increase in contact area between the hole transport layer and the ITO anode, and eventually increased the hole extraction efficiency in the organic solar cells (OSCs). The heterojunction OSCs fabricated on the ITO anode with a nano-scale surface roughness exhibited a higher power conversion efficiency of 3.320%, than that (2.938%) of OSCs made with the reference ITO/glass. The results here introduce a new method to improve the performance of OSCs by simply modifying the surface morphology of the ITO anodes.

Organic solar cells (OSCs) have been extensively investigated as next-generation eco-friend energy harvesting devices to replace Si-based solar cells because of some attractive features such as simple structure, printing-based simple process, light weight, low cost, and environmental friendly^1^,^2^,^3^,^4^,^5^. In spite of these attractive features, OSCs still require further investigation due to their low power conversion efficiency (PCE) relative to commercial Si-based or compound solar cells. In order to increase the PCE of OSCs, many aspects have been extensively investigated including synthesis donor/acceptor materials, design of the device architecture, use of optical spacers and improvement of optical and electrical properties of transparent electrodes^6^,^7^,^8^,^9^,^10^,^11^,^12^,^13^,^14^. For highly efficient OSCs, it is important to develop high-quality transparent electrodes because the short circuit current density (J_sc_) and fill factor (FF) of OSCs are closely related to an optical transmittance and a sheet resistance of the transparent electrodes^9^,^13^,^14^. The transparent electrodes with a higher optical transmittance can generate more excitons in the organic photoactive layer and increase the J_sc_ of OSCs^9^. In addition, a low sheet resistance of transparent electrodes can reduce total series resistance of the OSCs and increase of the FF value for OSCs^13^,^14^. Among several strategies that improve optical and electrical properties of transparent electrodes, surface modification of the electrode by using metal nanoparticles has been reported. For example, Reilly *et al.,* reported that Ag nano-hole films induced surface plasma enhanced photoconversion, they further suggested Ag nano-hole electrode as replacements for ITO anode^15^. Su *et al*. also reported that near-field enhancement of localized surface plasmons of Ag nanoparticles on an ITO electrode increased the PCE of their OSC by 17% relative to reference device^16^. Another effective way to improve the performance of devices is to control the surface morphology of the transparent electrode^17^,^18^,^19^. As discussed by Zhang *et al.,* light trapped in the ITO/organic wave-guided mode was efficiently extracted and light out-coupling was enhanced by using rougher ITO anodes for high-performance organic light emitting diodes^20^. Although intensive investigation of the surface modification of transparent electrodes or surface-plasmon related enhancement of PCE in OSCs has been carried out, there have been no reports on methods to improve the performance of OSCs using an ITO film with nano-scale surface roughness that was intentionally formed by self-segregated Ag nanoparticles embedded on the top region of the ITO films.

In this work, we report on a simple technique to make crystalline ITO (c-ITO) films with nano-scale surface roughness by removing self-segregated Ag nanoparticles in the surface grain boundary region of the c-ITO films. Electrical, optical, structural, and morphological properties of c-ITO films were investigated in detail as a function of the Ag-ITO mixed layer thickness. In addition, we fabricated conventional heterojunction OSCs on c-ITO to provide nano-scale surface roughness and we investigated its effect on the performance of OCSs. Finally, we investigated the interface between c-ITO anodes with nano-scale surface roughness and the hole transport layer using transmission electron microscopy (TEM).

## Results

**Figure 1:**
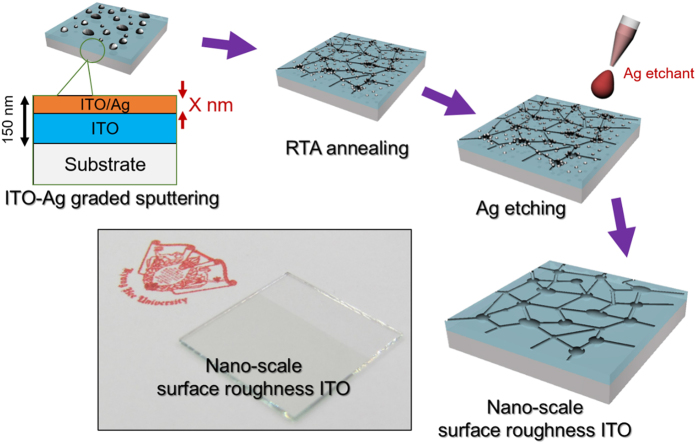
Schematic diagram illustrate the fabrication process of c-ITO with nano-scale surface roughness by removing Ag nanoparticles from the surface region of the c-ITO film. The picture shows highly transparent c-ITO films with a sheet resistance of 17 Ohm/square and an optical transparency of 86.8% at wavelength range of 400 to 800 nm.

Figure 1 shows a schematic diagram of the process used to make c-ITO films with nano-scale surface roughness on a glass substrate. After rapid thermal annealing, the samples were dipped into an iodine wet-etching solution to remove the self-segregated Ag nanoparticles in the grain boundaries and on the surface of the c-ITO. After removing the self-segregated Ag nanoparticles, the c-ITO films were cleaned using a conventional solution cleaning process. The total thickness of all ITO-Ag mixed layer/a-ITO samples was fixed at 150 nm. The a(c)-ITO films with different ITO-Ag mixed layer thicknesses were denoted as a(c)-ITO_148/2 nm_, a(c)-ITO_146/4 nm_, a(c)-ITO_144/6 nm_, and a(c)-ITO_142/8 nm_.

**Figure 2:**
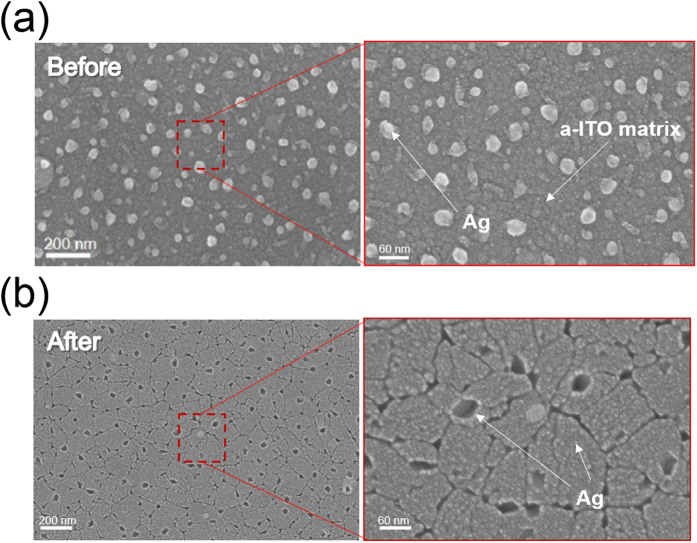
Surface FESEM images of the ITO_146/4nm_ films (**a**) before and (**b**) after rapid thermal annealing at 600 °C for 10 min. The as deposited sample showed agglomerated Ag islands on the surface of a-ITO matrix, while the annealed sample showed a crystallized ITO film with several nano-sized Ag islands and craters on the c-ITO surface.

Figure 2 shows surface FESEM images of the c-ITO_146/4nm_ films before and after rapid thermal annealing. The surface FESEM image of the as-deposited a-ITO_146/4nm_ sample in Fig. 2a showed the typical amorphous ITO surface and agglomerated Ag atoms on the top surface region of the ITO film. Although the ITO-Ag mixed layer was prepared by co-sputtering of the ITO and Ag targets at room temperature, randomly distributed Ag islands with sizes of 50 ± 10 nm were embedded in the a-ITO film. The Ag agglomeration on the ITO surface could be attributed to high surface energy of Ag metal^21^. The bright color in the enlarged FESEM image indicated by arrows illustrates the agglomerated Ag islands on the surface region of the a-ITO film. After rapid thermal annealing at 600 °C, the ITO_146/4nm_ film exhibited a completely different surface FESEM image from the as-deposited sample as shown in Fig. 2b. Annealing of the ITO_146/4nm_ film at 600 °C led to crystallization of the ITO film with a bixbyite structure and formation of a well-defined grain boundary. In addition, the c-ITO surface had several holes, which looked like meteorite craters. The enlarged image also showed self-segregated nano-sized Ag islands (bright color) covering the c-ITO surface as well as several large craters on the c-ITO surface. The formation of large holes on the c-ITO surface and the extinguished grain boundary could be attributed to rapid Ag diffusion from the large agglomerated Ag islands on the ITO surface to the grain boundary region of the c-ITO film during the rapid thermal annealing process. In general, the thermal annealing of the Ag film deposited on the oxide film led to the formation of nano-sized particles due to the high surface energy of the Ag atoms^21^. The nano-sized Ag islands observed on the as-deposited sample diffused into the grain boundary region of the c-ITO and created holes or widened the grain boundaries as shown in the high magnification FESEM image (Fig. 2b), because the activation energy of the grain boundary diffusion is much lower than that of lattice diffusion^22^.

**Figure 3:**
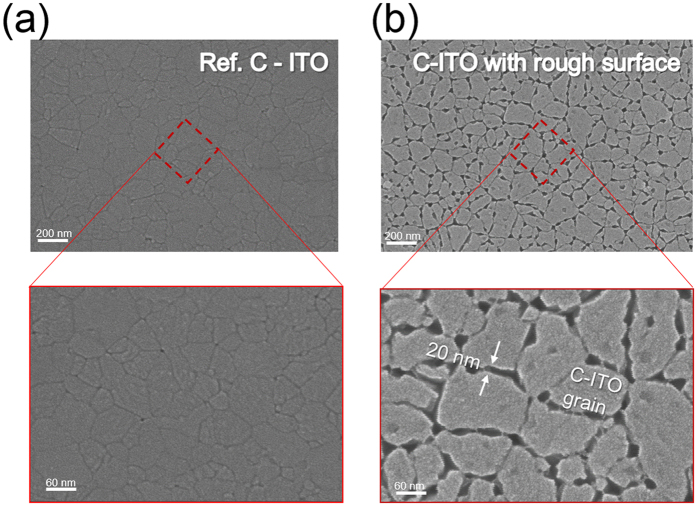
Surface FESEM images and enlarged image of the rapidly thermal annealed samples: (**a**) reference c-ITO film and (**b**) c-ITO film with nano-scale surface roughness after removing nano-sized Ag islands from the surface region of the c-ITO film.

Figure 3 shows a surface FESEM image of reference c-ITO and c-ITO films with nano-scale surface roughness after Ag wet-etching. The ITO film, annealed at 600 °C had a typical crystalline surface image with a smooth surface morphology, as shown in Fig. 3a 23. The enlarged image also shows well-defined grain boundaries of the c-ITO film, that do not have pinholes or cracks. However, the ITO_146/4nm_ film showed different surface morphologies after wet-etching of Ag nanoparticles as shown in Fig. 3b. The c-ITO films showed nano-scale surface roughness with a wide grain boundary because the Ag atoms on the surface of ITO or at the grain boundaries were completely removed using a wet etchant. Compared to the reference ITO film, the c-ITO in Fig. 3b showed a wider grain boundary between 10 and 30 nm. This wider grain boundary in the c-ITO film was easily filled in by coating with a PEDOT:PSS buffer layer and this increased contact area between the c-ITO and the PEDOT:PSS buffer layer.

**Figure 4:**
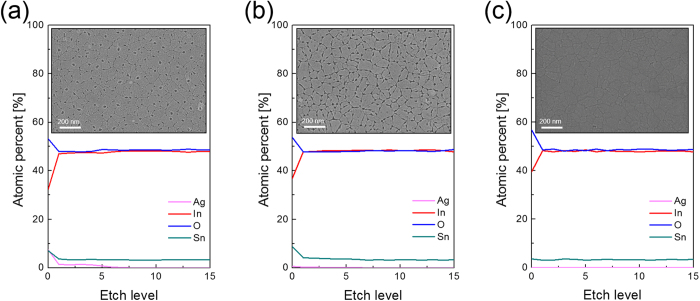
XPS depth profile of the c-ITO film with nano-scale surface roughness (**a**) before and (**b**) after dipping in the iodine solution. The inset shows the surface FESEM images. (**c**) XPS depth profile of reference c-ITO films.

Figure 4a,b exhibit the XPS depth profile of a c-ITO film with nano-scale surface roughness before and after Ag wet-etching from the grain boundaries and surface of the c-ITO films. The c-ITO film before wet-etching showed constant In, Sn, and O content in atomic percent with increasing etch levels, indicating uniformly doped Sn dopant was present in the In_2_O_3_ matrix (Fig. 4a). In addition, Ag atoms were observed at the top region of the c-ITO film due to diffusion of Ag through the grain boundary of the c-ITO film during rapid thermal annealing. After wet-etching of segregated Ag atoms, the c-ITO film with nano-scale surface roughness showed only In, Sn and O elements, which is similar to the XPS depth profile of a reference c-ITO film as shown in Fig. 4b,c. Identical XPS depth profile results indicated the effective removal of self-segregated Ag atoms from the grain boundary and surface of the c-ITO film.

**Figure 5:**
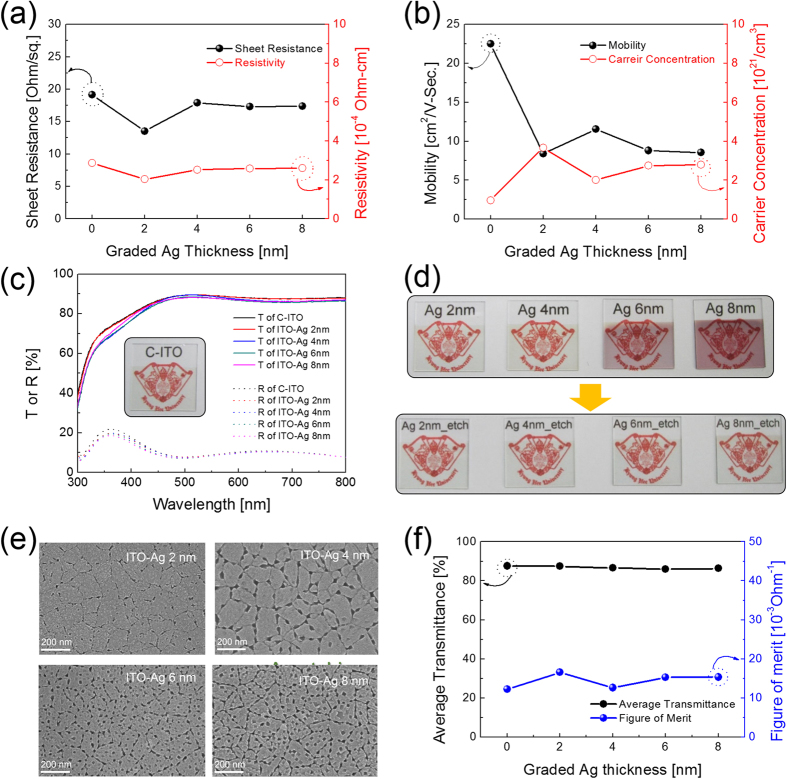
(**a**) Resistivity and sheet resistance and (**b**) mobility and carrier concentration of the c-ITO film with a nano-scale surface roughness as a function of ITO-Ag mixed layer thickness. (**c**) Diffusive transmittance and reflectance of the c-ITO film with nano-scale surface roughness. The inset picture shows the transparency of the reference c-ITO. (**d**) Pictures of c-ITO films with nano-scale surface roughness before and after Ag wet-etching. (**e**) Surface FESEM images of c-ITO films with nano-scale surface roughness with increasing ITO-Ag mixed layer thickness. (**f**) Figure of merit value for the c-ITO film with nano-scale surface roughness with increasing ITO-Ag mixed layer thickness.

Figure 5a,b shows Hall measurement results of c-ITO films with nano-scale surface roughness as a funciton of ITO-Ag mixed layer thickness after wet-etching of self-segregated Ag atoms. Regardless of the ITO-Ag mixed layer thickness, the c-ITO films with nano-scale surface roughness showed sheet resistances (15 ± 2 Ohm/square) and resistivities (2.3 ± 0.3 × 10^−4 ^Ohm-cm) similar to reference c-ITO film as shown in Fig. 5a. We attributed these results to the very thin thickness of the ITO-Ag mixed layer region and effective removal of Ag atoms. Fig. 5b shows the mobility and carrier concentration of the c-ITO films with nano-scale surface roughness as a function of ITO-Ag mixed layer thickness. During rapid thermal annealing, some Ag atoms formed a crystalline silver oxide phase (Ag_2_O). Subrahmanyam *et al.,* who investigating the characteristics of Ag-doped In_2_O_3_ films, reported that oxygen in the In_2_O_3_ matrix reacts more with Ag than with In elements^24^. The formaiton of a Ag_2_O phase during rapid thermal annealing led to additional oxygen vacancies, which generated excess electrons. This observation explains why the carrier concentration of the c-ITO films with nano-scale surface roughness was higher than that of the reference c-ITO film. However, carrier mobility of the c-ITO with nano-scale surface roughness was slightly lower than that of the reference c-ITO film due to scattering of carriers at the rough surface. Consequently, the sheet resistance and resistivity of c-ITO films with nano-scale surface roughness were similar to those of the reference c-ITO film due to their low mobility and high carrier concentration. Figure 5c shows the diffusive transmittance and reflectance of the c-ITO films with nano-scale surface roughness as a function of the ITO-Ag mixed layer thickness and reference c-ITO film. All c-ITO films with nano-scale surface roughness demonstrated a similar high diffusive transmittance in the visible wavelength range (400 to 800 nm) regardless of the ITO-Ag mixed layer thickness even though it has a slightly different surface morphology. The average transmittance between 400 and 800 nm and the diffusive transmittance at 550 nm are summarized in Table 1 for the c-ITO films with nano-scale surface roughness. The diffusive transmittance (86.6%) of the c-ITO film with nano-scale surface roughness was similar to the reference c-ITO film (87.6%). Figure 5d shows a picture of c-ITO films with nano-scale surface roughness before and after wet-etching. Before wet-etching, the samples were a different color due to the different ITO-Ag mixed layer thicknesses. However, after wet-etching of Ag atoms from the grain boundary and surface of the c-ITO films, all samples show optical transparency similar to the reference c-ITO film (Inset of Fig. 5c), regardless of the ITO-Ag mixed layer thickness. As shown, the symbol of Kyung Hee University is clearly seen through the c-ITO films even though they had nano-scale rough surface. The reflectance of the reference c-ITO and c-ITO film with nano-scale surface roughness were similar at 550 nm and between 400 nm and 800 nm. However, for wavelengths between 300 and 450 nm, the diffusive transmittance of the c-ITO film with nano-scale surface roughness was slightly lower than that of the reference c-ITO due to scattering effects on the rough surface. In addition, the different shapes and size of the nano-scale surface roughness on the ITO film may affect the scattering of light of the c-ITO films. Thus, reflectance of c-ITO films with rough surfaces decreased with increasing ITO-Ag mixed layer thickness. Figure 5e exhibits the surface FESEM images of the c-ITO film with nano-scale surface roughness with increasing ITO-Ag mixed layer thickness. As shown, the increase in the ITO-Ag mixed layer thickness led to increased surface roughness of the c-ITO film because more Ag atoms segregated to the grain boundaries. Wet-etching of different amounts of Ag atoms in the grain boundary resulted in a different surface morphology. Therefore, nano-scale surface roughness of the c-ITO film was controlled by adjusting the thickness of the ITO-Ag mixed layer. The figure of merit (FOM, T^10^/R_s_) was calculated based on the measured sheet resistance (R_s_) and transmittance (T) of c-ITO films with nano-scale surface roughness, as a function of ITO-Ag mixed layer thickness as shown in Fig. 5f. Due to the similar sheet resistance and diffusive transmittance, all c-ITO films with nano-scale surface roughness showed similar FOM values to that of the reference c-ITO film. Therefore, we concluded that the existence of nano-scale surface roughness did not affect the electrical and optical properties of the c-ITO film if we completely removed the self-segregated Ag atoms.

**Figure 6:**
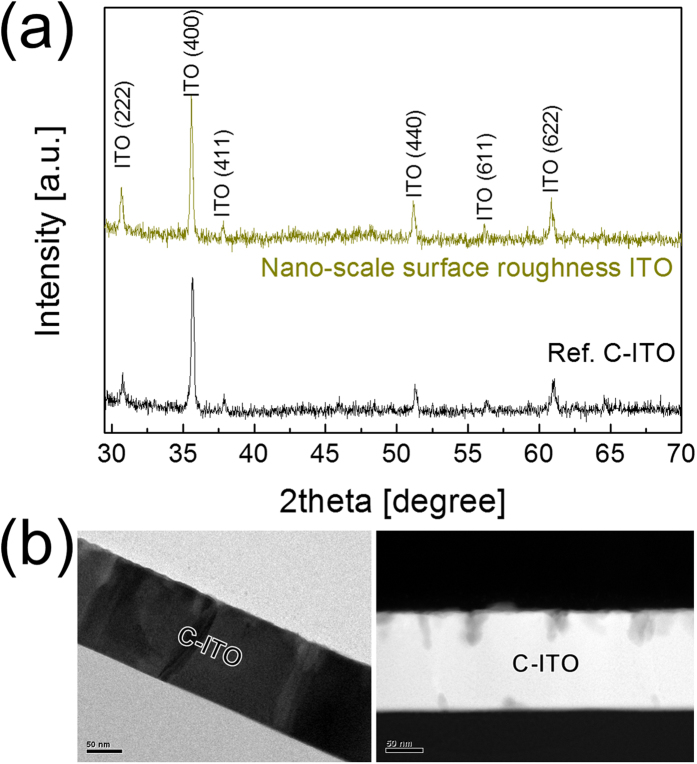
(**a**) XRD plots obtained from c-ITO with nano-scale surface roughness and reference c-ITO film. (**b**) Bright field (BF) and dark field (DF) TEM images obtained from the c-ITO film with nano-scale surface roughness. The DF TEM image reveals a number of dark blobs, which are distributed on the surface of the c-ITO film with nano-scale surface roughness.

Figure 6a shows XRD plots obtained from c-ITO with nano-scale surface roughness and reference c-ITO films. Both samples showed a strong (400) peak at 2θ = 35.56°, which was unlike typical ITO films with a (222) preferred orientation. The strong (400) peak of the c-ITO film with nano-scale surface roughness indicated that the c-ITO film had a columnar structure perpendicular to the glass substrate. The strong (400) preferred orientation was attributed to the substrate temperature and annealing conditions^25^,^26^. In addition, both films showed crystalline (222), (411), (440), (611) and (622) peaks at 2θ = 30.68°, 37.82°, 51.14°, 56.16°, and 60.88°, indicating a typical bixbyite structure. Using Debye-Scherrer equation^27^,^28^,^29^, the crystalline sizes of c-ITO films with nano-scale surface roughness were calculaed. The crystalline sizes of the c-ITO with a preffered orienation of (222), (400), (411), (440), (611), and (622) are 41.81, 42.01, 42.08, 47.29, 61.12, and 33.08 nm, respectively. The reference c-ITO films also showed similar crystalline sizes becasue small amount of Ag atoms on the surface region could not influence on the crystalline sizes of ITO films. Furthermore, the XRD plot of c-ITO with nano-scale surface roughness showed no crystalline Ag peak, which indicates the effective removal of self-segregated Ag atoms from the surface and grain boundary of the c-ITO film. Figure 6b shows bright field (BF) and dark field (DF) cross-sectional TEM images obtained from a c-ITO film with nano-scale surface roughness. It is difficult to observe the nano-scale suface roughness on the c-ITO film in BF cross-sectional TEM image. However, the DF TEM image clearly showed the existence of a surface roughness layer, as observed in the surface FESEM image in Fig. 3b. In the surface region of the c-ITO film with nano-scale surface roughness, there is a dark contrast, which we belive to be widened grain boundary or holes lefting after removal of the Ag atoms by the wet etchant.

**Figure 7:**
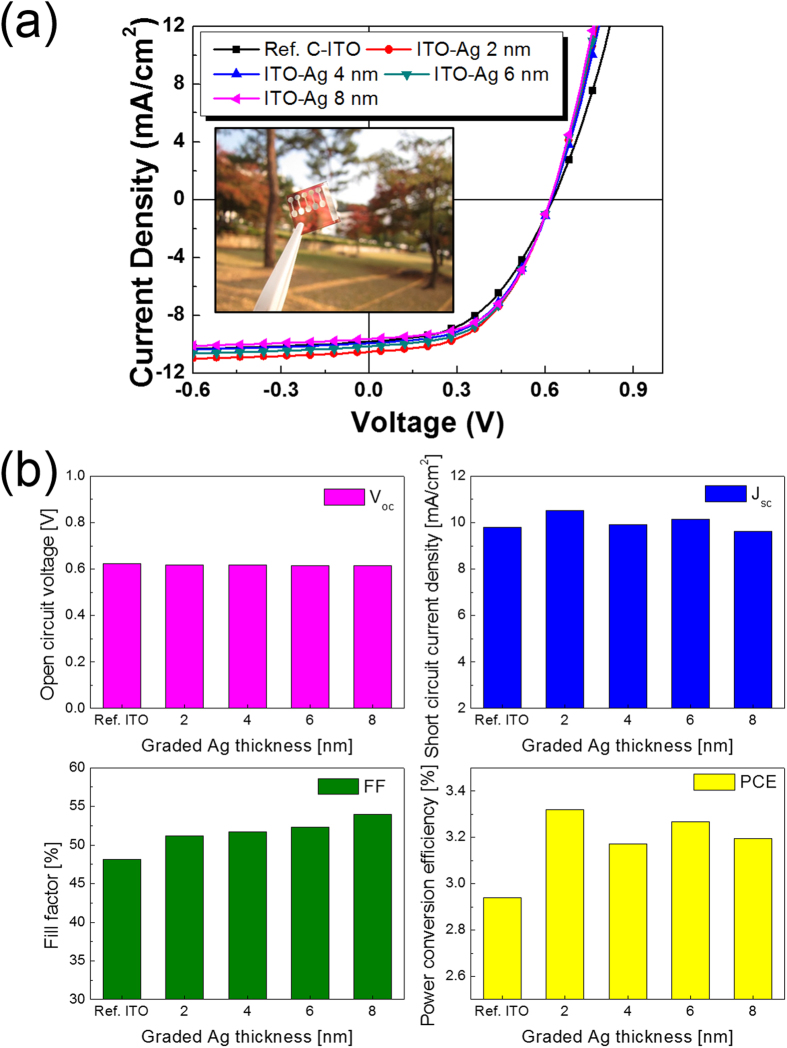
(**a**) J-V curves of OSCs fabricated on c-ITO with nano-scale surface roughness as a function of ITO-Ag mixed layer thickness. Inset is a picture of OSC fabricated on c-ITO with optimized nano-scale surface roughness. (**b**) Main performance parameter of OSCs including open circuit voltage, short circuit current density, fill factor, and power conversion efficiency with increasing ITO-Ag mixed layer thickness.

Figure 7a shows the *J-V* curves for the OSCs fabricated on the c-ITO films with nano-scale surface roughness with increasing ITO-Ag mixed layer thicknesses. Detailed related to the performance of the OSCs are summarized in Table 2. It was noteworthy that the power conversion efficiency of the OSC fabricated on the c-ITO anode with nano-scale surface roughness was higher than that of the OSC with the reference c-ITO even though both c-ITO films had a similar sheet resistance and optical transmittance. Figure 7b shows the main parameters of the OSCs including the open-circuit voltage (V_oc_), short circuit current density (J_sc_), fill factor (FF), and power conversion efficiency (PCE) obtained from the J-V curves as a function of ITO-Ag mixed layer thickness. The V_oc_ values of all OSCs were between 0.615 and 0.632 V, indicating that the V_oc_ value of the OSCs was not significantly affected by the ITO-Ag mixed layer thickness due to the presence of the PEDOT:PSS buffer layer, which was used on the top surface of the c-ITO anode. However, the J_sc_ value of the OSCs with c-ITO films with nano-scale surface roughness was slightly higher than that of the OSC with reference c-ITO anode due to the increased contact area and scattering of light on the rough surface. The FF values of the OSCs fabricated with rough c-ITO anode increased with increasing ITO-Ag mixed layer thickness. The FF value of the OSC was mainly affected by the series resistance in the OSCs. In general, the series resistance (R_series_) of OSCs can be expressed as follows.
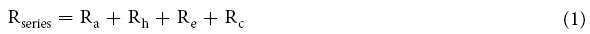


Here, R_a_ is the resistance of organic active layer, R_h_ is the resistance of the hole transport layer, R_e_ is the resistance of the electrodes and R_c_ is the contact resistance between each layer^30^. Because all organic layers and metal cathode layers were coated on the reference c-ITO and rough c-ITO anodes under identical conditions, the only difference in the OSCs was the interface region between the anode and the PEDOT:PSS buffer layer. Therefore, the contact resistance between the anode and the hole transport layer was the main parameter affecting the FF value of the OSCs. As shown in Fig. 5e, the increase in the ITO-Ag mixed layer thickness led to an increase in contact area between the anode and the hole transport layer. Increased contact area by nano-scale roughness could increase the hole extraction from the PEDOT:PSS buffer layer. Na *et al*., investigating the effect of contact area on the performance of GaN-based light emitting didoes, reported that increased contact area between the transparent electrode and p-GaN led to a reduction in the contact resistance of the ohmic layer^31^. Therefore, the lower series resistance and higher FF value for the OSC fabricated on the c-ITO anode with nano-scale surface roughness was attributed to the decreased contact resistance^32^. The PCEs of the OSCs made with rough c-ITO were also affected by the ITO-Ag mixed layer thickness because the FF values depend on the ITO-Ag mixed layer thickness. As a result, the OSC for the ITO_148/2nm_ film showed the highest PCE of 3.32%, which is 11.5% higher than the reference OSC. Therefore, we suggested that the c-ITO with nano-scale surface roughness is a promising transparent anode for high performance OSCs.

**Figure 8:**
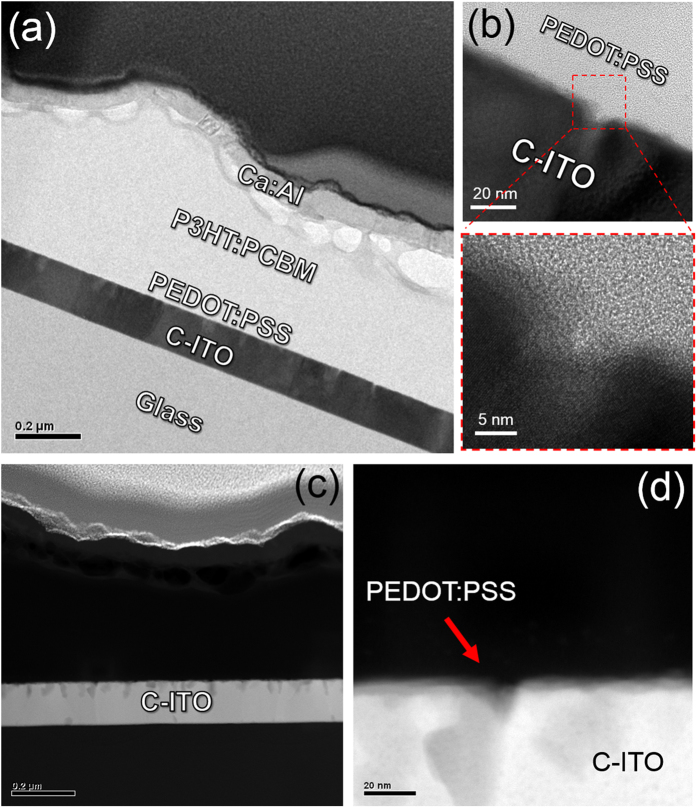
(**a**) BF cross-sectional TEM image and (**b**) enlarged HRTEM images of OSCs fabricated on c-ITO anode with nano-scale surface roughness. (**c**) The DF cross-sectional TEM image and (**d**) enlarged HRTEM image of OSCs based on c-ITO with nano-scale surface roughness.

HRTEM examination was carried out to investigate the microstructure and interface structure of OSCs fabricated on a c-ITO_146/4nm_ anode with nano-scale surface roughness. Figure 8a shows a BF cross-sectional TEM image of OSC, which showed a well-distinguished ITO anode, organic layer, and metal cathode layer. Even though the surface of the c-ITO anode was rough, the interface between the PEDOT:PSS and ITO was very clear and smooth. Enlarged image in Fig. 8b, shows that the PEDOT:PSS buffer layer filled out the widened grain boundary region of the c-ITO anode because the PEDOT:PSS was spin-coated from solution. The DF TEM images in Fig. 8c,d show that the PEDDOT:PSS buffer layer filled out the nano-scale rough surface of the c-ITO anode and provided good contact with the c-ITO anode. Thus, the contact area between c-ITO anode and PEDOT:PSS layer increased. This allowed more hole carriers to be extracted from the PEDOT:PSS layer to the anode and increased the PCE of the OSCs.

## Conclusion

We investigated the characteristics of c-ITO films with nano-scale surface roughness for use as transparent anodes for high-performance OSCs. Nano-scale surface roughness in the c-ITO film was artificially formed by embedding and wet-etching of self-segregated Ag nanoparticles on the top of the c-ITO film to increase hole extraction efficiency of the OSCs. Effective removal of self-segregated Ag particles in the grain boundary of the ITO film led to an ITO film with nano-scale surface roughness having a sheet resistance of 17.8 Ohm/square and an optical transmittance of 86.6%. Nano-scale surface roughness of the ITO film resulted in an increase in contact area between the hole transport layer and ITO anode, which eventually increased the hole extraction efficiency of the OSCs. The bulk heterojunction OSCs fabricated on the c-ITO anode with nano-scale surface roughness exhibited an improved power conversion efficiency of 3.320%, relative to that (2.938%) of reference OSC. Therefore, we demonstrated that ITO films with nano-scale surface roughness were more suitable for high-performance OSCs as transparent anodes.

## Methods

### Graded sputtering of ITO and Ag films

To fabricate c-ITO films with nano-scale surface roughness, an ITO and Ag graded sputtering technique was employed, as we described previously^33^,^34^. A direct current (DC) magnetron sputtering system with tilted multi-cathode guns was used for graded sputtering as illustrated in Figure S1. First, amorphous ITO (a-ITO) was sputtered on 15 × 15 mm^2^ glass substrate using a 3 inch ITO ceramic target at room temperature. The a-ITO film was prepared at a constant DC power of 100 W, a working pressure of 2 mTorr, an Ar/O_2_ flow ratio of 20/0.3 sccm, and a substrate rotation rate of 20 rpm. Then, the ITO-Ag mixed layer was directly sputtered onto the top a-ITO layer by simultaneous sputtering of Ag metal and ITO ceramic targets to provide an ITO-Ag mixed layer with thicknesses ranging from 2 to 8 nm. A DC power of 20 W was applied to the 3 inch Ag metal target. The total thickness of all ITO-Ag mixed layer/a-ITO samples was fixed at 150 nm.

### Formation of nano-scale rough ITO

After deposition of the a-ITO films covered with ITO-Ag mixed layer as a function of thickness, all samples were rapidly thermal annealed under vacuum for 10 min at 600 °C to crystallize the ITO film and form self-segregated Ag nanoparticles on the surface region of the c-ITO films. After rapid thermal annealing, the samples were dipped into an iodine wet-etching solution (AN-50, Solaronix) for 10 min to remove the self-segregated Ag nanoparticles in the grain boundaries and on the surface of the c-ITO. After removing the self-segregated Ag nanoparticles, the c-ITO films were cleaned using a conventional solution cleaning process.

### Analysis of c-ITO films with nano-scale surface roughness

Electrical properties of the c-ITO films with nano-scale surface roughness were investigated using Hall measurement system (HL5500PC, Accent Optical Technology) as a function of ITO-Ag mixed layer thickness. Optical transmittances of the c-ITO films with nano-scale surface roughness were measured using a UV/visible spectrometer (UV 540, Unicam). The surface morphology of the c-ITO films with nano-scale surface roughness before and after the rapid thermal annealing process were examined by field emission scanning electron microscopy (FESEM, LEO SUPRA 55). In addition, the structure of the films was evaluated by X-ray diffraction (XRD, D/Max 2500, Rigaku) using Cu Kα radiation (λ = 0.154 nm). X-ray photoelectron spectroscopy (XPS, Thermo Fisher Scientific Co. Ltd.) with an Al Kα X-ray source was used to analyze the content of Ag on the ITO films before and after wet-etching. In addition, the microstructure of the films and the interfacial structure of the OSCs were examined using high resolution transmission electron microscopy (HRTEM: JEM-2100F). Cross-sectional samples were prepared for HRTEM examination by focused ion beam milling.

### Fabrication of heterojunction organic solar cells

To investigate the feasibility of the c-ITO films with nano-scale surface roughness as transparent anodes for OSCs, we fabricated conventional heterojunction OSCs on the c-ITO films with nano-scale surface roughness. Prior to coating the films with poly(3,4-ethylenedioxythiophene) polystyrene sulfonate (PEDOT:PSS), the surface of the c-ITO films was irradiated by UV-ozone for 20 min to remove carbon-based contamination and improve the adhesion of the PEDOT:PSS layer. Commercially available PEDOT:PSS ink (Clevios AI4083) was spin-coated on the c-ITO films and was then annealed at 110 °C for 10 min to remove solvent. After applying the PEDOT:PSS layer coating, a blend solution of 30 mg of poly(3-hexylthiophene) (P3HT) (Rieke Metals) and 15 mg of Phenyl-C_61_-butyric acid methyl ester (PCBM) (Nano-C) in 2 ml of chlorobenzene was spin-coated on the PEDOT:PSS layer under a nitrogen atmosphere. The resulting film was annealed at 110 °C for 10 min. Finally, a Ca/Al (20 nm/100 nm) cathode with an area of 4.66 mm^2^ was evaporated onto the P3HT:PCBM layer. By using a thin metal shadow mask, a dumbbell-shaped cathode electrode was patterned onto the P3HT:PCBM layer. Figure S2 illustrated schematic fabrication process of heterojunction OSCs on the c-ITO with nano-scale surface roughness. The photocurrent density-voltage (*J-V*) characteristics of the OSCs fabricated on the c-ITO anode with nano-scale surface roughness were investigated by using a Keithley 1200 source measure unit under 100 mW/cm^2^ illumination with AM 1.5 G irradiation produced by a solar simulator. In order to measure performance of OSCs accurately, a solar simulator (SoI3A Class AAA, Oriel Instruments, USA) was calibrated precisely by using the standard Si solar cell (SRC-1000-TC-KG5-N, VLSI standards, Inc., USA) guaranteed from the National Renewable Energy Laboratory (NREL).

## Additional Information

**How to cite this article**: Kim, H.-J. *et al*. Tin doped indium oxide anodes with artificially controlled nano-scale roughness using segregated Ag nanoparticles for organic solar cells. *Sci. Rep.* **6**, 33533; doi: 10.1038/srep33533 (2016).

## Supplementary Material

Supplementary Information

## Figures and Tables

**Table 1:** Diffusive transmittance and reflectance of c-ITO film with nano-scale surface roughness as a function of Ag-ITO mixed layer thickness.

	Graded Ag thickness	Ref. c-ITO	2 nm	4 nm	6 nm	8 nm
Transmittance	At 550 nm	89.09	89.09	88.81	87.84	87.83
	Average (400~800 nm)	87.6	87.4	86.6	85.9	86.3
Reflectance	At 550 nm	8.79	8.54	8.33	8.41	8.46
	Average (400~800 nm)	9.96	9.65	9.62	9.44	9.34

**Table 2:** Performance of the OSCs fabricated on ITO/glass substrates with nano-scale surface roughness and reference c-ITO/glass substrates.

OSC structure	V_oc_ [V]	J_sc_ [mA/cm^2^]	FF [%]	R_series_ [Ω]	PCE [%]
Ref c-ITO	0.632	9.798	48.12	380	2.938
ITO_148/2nm_	0.617	10.505	51.22	280	3.320
ITO_146/4nm_	0.619	9.909	51.72	283	3.172
ITO_144/6nm_	0.616	10.140	52.30	280	3.266
ITO_142/8nm_	0.615	9.629	53.94	272	3.196
