# Supplementary material for: Tin doped indium oxide anodes with artificially controlled nano-scale roughness using segregated Ag nanoparticles for organic solar cells
Source: Sci Rep. 2016 Sep 19;6:33533. doi: 10.1038/srep33533 (PMC5027522; doi:10.1038/srep33533)
Supplement: Supplementary Information [file srep33533-s1.pdf]

Supporting Information

Tin doped indium oxide anodes with artificially controlled nano-scale roughness using segregated Ag nanoparticles for organic solar cells

Hyo-Joong Kim[1], Eun-Hye Ko[1], Yong-Jin Noh[2], Seok-In Na[2], and Han-Ki Kim[1,*]

[1]Kyung Hee University, Department of Advanced Materials Engineering for Information and Electronics, 1 Seocheon, Yongin, Gyeonggi-do 446-701, Republic of Korea
[2]Chonbuk National University, Graduate School of Flexible and Printable Electronics, 664-14, Deokjin-dong, Jeonju-si, Jellabuk-do, 561-756, Republic of Korea
[*]imdlhkkim@khu.ac.kr

To make segregated Ag nanoparticles on ITO films, we employed a graded sputtering technique as shown in upper pannels of **Figure S1**. First, the ITO film was sputtered on a glass substrate at a constrant DC power of 100 W. And then, the Ag-ITO mixed layer was formed on the surface region of the ITO flm by graded sputtering of metal Ag and ceramic ITO targest at different DC power of 100 W and 25 W as shown in **Figure 1S**

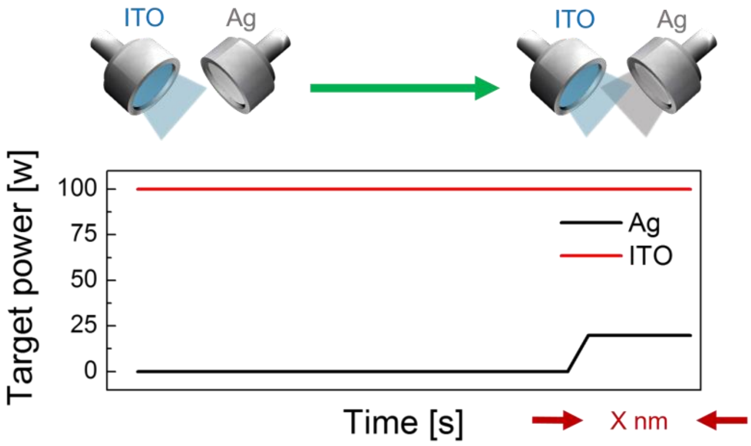

Figure S1. Schematics of Ag and ITO graded sputtering. DC power applied to Ag and ITO target as a function of sputtering time to fabricate ITO electrode covered by Ag-ITO mixed layer. Upper panels showed the plasma formed on ITO and Ag targets.

The thickness (X nm) of the Ag-ITO mixed layer is a key parameter to control the size of Ag nanoparticles and morphology of the ITO films with nano-scale surface roughness.

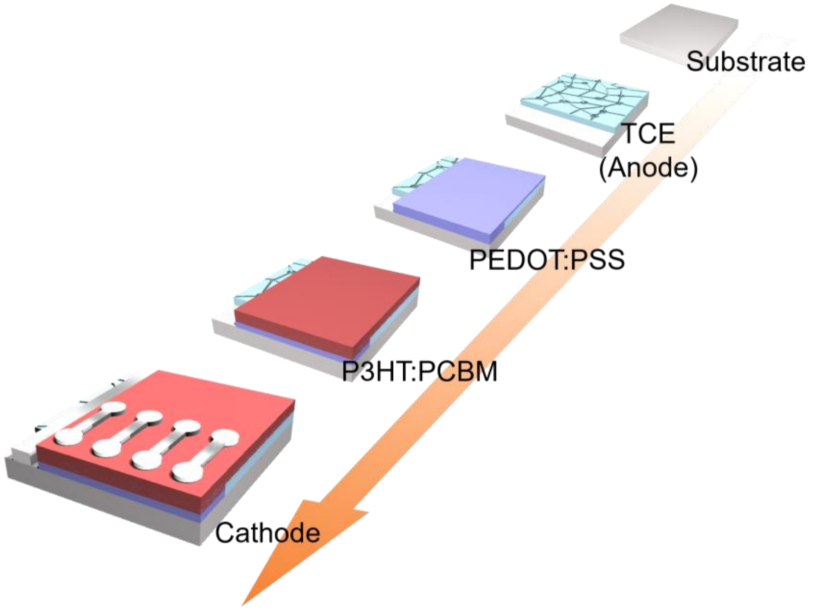

Figure S2. Fabrication of heterojunction OSC on c-ITO with nano-scale surface roughness.
